# Heme oxygenase-1 derived carbon monoxide suppresses A*β*_1–42_ toxicity in astrocytes

**DOI:** 10.1038/cddis.2017.276

**Published:** 2017-06-15

**Authors:** Nishani T Hettiarachchi, John P Boyle, Mark L Dallas, Moza M Al-Owais, Jason L Scragg, Chris Peers

**Affiliations:** 1 Division of Cardiovascular and Diabetes Research, LICAMM Faculty of Medicine and Health, University of Leeds, Leeds LS2 9JT, UK; 2 Reading School of Pharmacy, University of Reading, Reading, RG6 6UB, UK

## Abstract

**Neurodegeneration in Alzheimer’s disease (AD) is extensively studied, and the involvement of astrocytes and other cell types in this process has been described. However, the responses of astrocytes themselves to amyloid *β* peptides ((A*β*; the widely accepted major toxic factor in AD) is less well understood. Here, we show that A*β*_(1-42)_ is toxic to primary cultures of astrocytes. Toxicity does not involve disruption of astrocyte Ca^2+^ homeostasis, but instead occurs via formation of the toxic reactive species, peroxynitrite. Thus, A*β*_(1-42)_ raises peroxynitrite levels in astrocytes, and A*β*_(1-42)_ toxicity can be inhibited by antioxidants, or by inhibition of nitric oxide (NO) formation (reactive oxygen species (ROS) and NO combine to form peroxynitrite), or by a scavenger of peroxynitrite. Increased ROS levels observed following A*β*_(1-42)_ application were derived from NADPH oxidase. Induction of haem oxygenase-1 (HO-1) protected astrocytes from A*β*_(1-42)_ toxicity, and this protective effect was mimicked by application of the carbon monoxide (CO) releasing molecule CORM-2, suggesting HO-1 protection was attributable to its formation of CO. CO suppressed the rise of NADPH oxidase-derived ROS caused by A*β*_(1-42)_. Under hypoxic conditions (0.5% O_2_, 48 h) HO-1 was induced in astrocytes and A*β*_(1-42)_ toxicity was significantly reduced, an effect which was reversed by the specific HO-1 inhibitor, QC-15. Our data suggest that A*β*_(1-42)_ is toxic to astrocytes, but that induction of HO-1 affords protection against this toxicity due to formation of CO. HO-1 induction, or CO donors, would appear to present attractive possible approaches to provide protection of both neuronal and non-neuronal cell types from the degenerative effects of AD in the central nervous system.**

The progression of Alzheimer’s disease (AD), from early loss of functional synapses^1, 2, 3^ to the loss of neurones through apoptosis and other pathways^4, 5, 6, 7^ has been described extensively, yet remains to be fully understood. The association of disease progression with increased levels of amyloid *β* peptide (A*β*; predominantly the 1–42 form, A*β*_1–42_) is also well established: this peptide is neurotoxic, and is also an important constituent of disease-characterising plaques.^8, 9^

Astrocytes are as numerous as neurons in the central nervous system^10^ and their role in neurodegenerative diseases has also been extensively explored.^11, 12, 13^ These cells are diverse in structure and function, regulating CNS homeostasis in general and shaping activity at individual synapses.^11, 14, 15^ More recently there is a growing appreciation of the heterogeneity of astrocytes.^16^ In AD, and transgenic murine models of AD, atrophy of astrocytes is widely reported, and reactive astrocytes (in part defined as staining positively for glial fibrillar acidic protein; GFAP) have long been anatomically associated with plaques (reviewed in ref. 11). Atrophic loss of morphology clearly undermines the ability of astrocytes to regulate synaptic activity, and disrupts the ‘neurovascular unit’ in which astrocytes play a central role in balancing local blood flow to neuronal activity.^17, 18^

Numerous *in vitro* studies have also established that astrocytes can, at least in part, mediate the neurotoxic effects of A*β*. Thus, for example, extrasynaptic glutamate release from astrocytes in response to A*β* exposure leads to synaptic damage and loss.^19^ Evidence exists that suggests A*β* disrupts astrocyte [Ca^2+^]_i_ and by doing so activates reactive oxygen species (ROS) production by NADPH oxidase (reviewed in ref. 20). This increases lipid peroxidation in both astrocytes and neurones, which in turn depletes glutathione (GSH) levels in both cell types. Since neurones require delivery of GSH precursors from astrocytes, they are preferentially susceptible to continued oxidative stress and so die, whereas astrocytes have a greater antioxidant capacity to survive. It should be noted, however, that this model is not universally accepted, and others have indicated that astrocytic Ca^2+^ signalling is not disrupted by A*β*, at least over the same timecourse, and that different downstream signalling pathways are evoked.^21, 22^

Despite the extensive literature on the involvement of astrocytes in the progression of neurodegeneration in AD, information available concerning the molecular mechanisms underlying responses of astrocytes themselves to A*β* is relatively limited and appears seemingly contradictory in some respects. Thus, for example, astrocyte viability has been reported to be unaffected or even slightly potentiated following exposure to sub-micromolar A*β*_1-42_ for 24 h.^23^ By contrast, others have shown that astrocytes are susceptible to micromolar A*β*−induced death over similar time periods.^24^ In the present study, we demonstrate that cortical astrocytes can undergo apoptotic death when exposed to sub-micromolar levels of A*β*_1-42_, and that this occurs via formation of peroxynitrite (ONOO^–^). Furthermore, we demonstrate a complex pattern of modulation of this process of A*β* toxicity by the induction of the antioxidant enzyme haem oxygenase-1 (HO-1).

## Results

**Figure 1:**
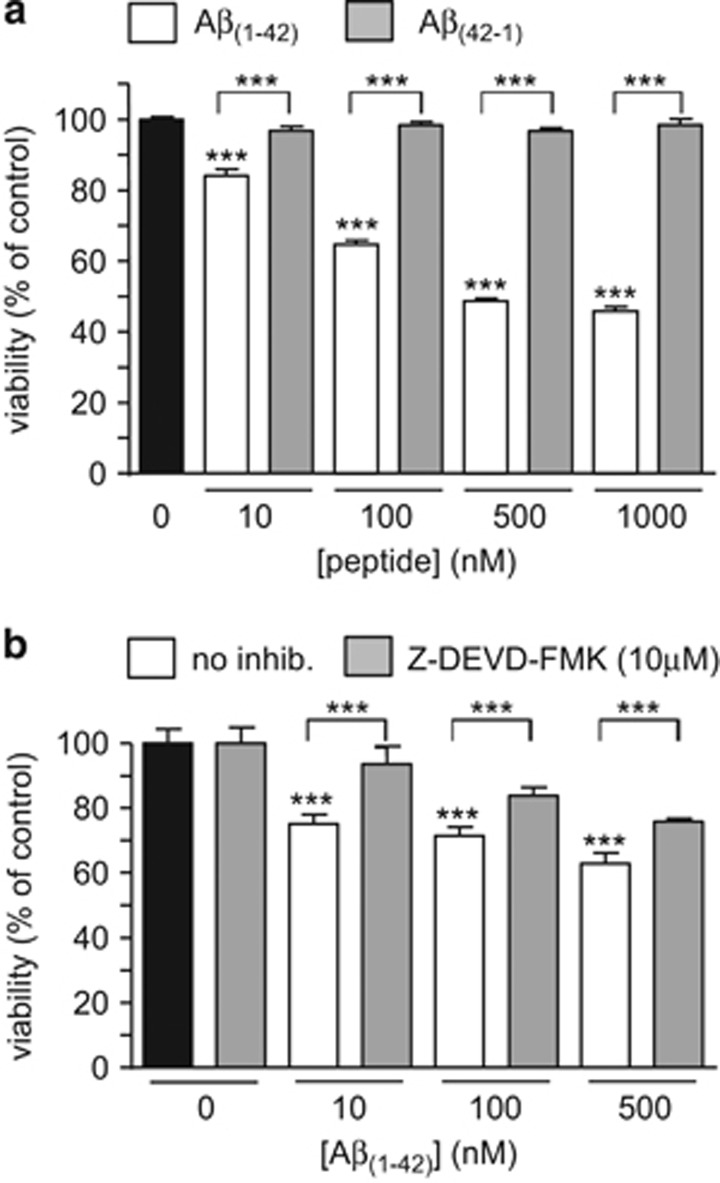
Amyloid peptide A*β*_1–42_ is toxic to astrocytes in part via inducing apoptosis. (**a**) Effect on cell viability of a 24 h exposure of astrocytes to A*β*_1–42_ (10–1000 nM, white bars) and the reverse peptide A*β*_42-1_ (grey bars) using the mitochondrial activity-based MTT assay. Bars represent the mean±S.E.M. data of cells from 4 repeats (each performed in duplicate) with cells from different preparations. (**b**) as (**a**), except cells were exposed either to A*β*_1–42_ alone (10–500 nM, white bars) or A*β*_1–42_ in the additional presence of 10 *μ*M Z-DEVD-FMK, a caspase-3 inhibitor (grey bars). Bars represent the mean±S.E.M. data of cells from 3 repeats (each performed in duplicate) with cells from different preparations. Significant difference: ***P<0.001 effects of peptide alone compared to control, or between amyloid peptide and reverse peptide (**a**), or effects of Z-DEVD-FMK at each amyloid concentration, as indicated

Exposure of astrocytes to A*β*_1-42_ (10 nM-1 *μ*M) for 24 h caused a concentration-dependent loss of viability, as shown in Figure 1a. This toxic effect of A*β* appeared selective, since the reverse sequence peptide, A*β*_42-1_, was without effect over the same concentration range (Figure 1a). In the presence of the caspase-3 inhibitor Z-DEVD-FMK (10 *μ*M) the toxic effects of A*β*_1–42_ were partially reversed, suggesting the toxicity of A*β*_1–42_ was at least partly due to the induction of apoptosis.

**Figure 2:**
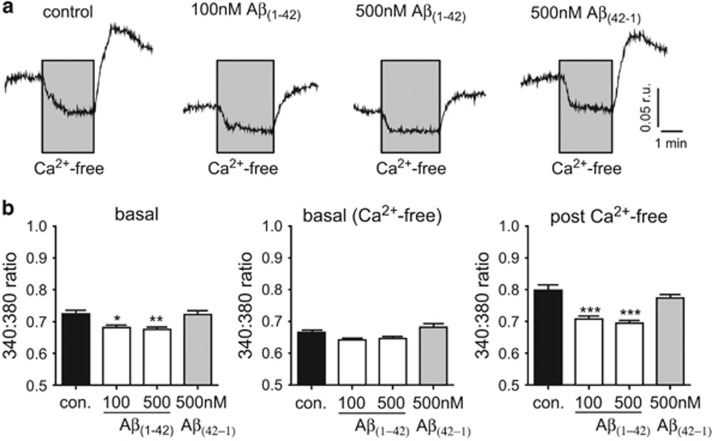
A*β*_1–42_ does not dramatically alter [Ca^2+^]_i_ in astrocytes. (**a**) Example microfluorimetric measurements of [Ca^2+^]_i_ in astrocytes under control conditions, or exposed to A*β*_1–42_ or A*β*_42-1_ as indicated, for 24 h. Scale bars apply to all traces. In each case, for the period indicated by the grey area, extracellular Ca^2+^ was replaced with 1mM EGTA. (**b**) Mean±S.E.M. levels of [Ca^2+^]_i_ measured in 8–9 recordings under normal conditions (left), during exposure to Ca^2+^-free solution (containing 1 mM EGTA; middle) and following replacement of Ca^2+^ in the perfusate (right). Significance: *P<0.05; **P<0.01; ***P<0.001 as compared with controls

Since it has previously been suggested that amyloid peptides disrupt [Ca^2+^]_i_ in astrocytes, we next examined whether [Ca^2+^]_i_ was altered in astrocytes at the levels we found to be toxic. As shown by the examples in Figure 2a, and the mean data of Figure 2b, exposure of astrocytes to 100 nM or 500 nM A*β*_1–42_ for 24 h caused a modest but significant reduction in resting [Ca^2+^]_i_ whereas 500 nM reverse peptide (A*β*_42-1_) was without significant effect. Removal of extracellular Ca^2+^ (replaced with 1 mM EGTA) caused a reversible fall of [Ca^2+^]_i_ in all cell groups examined, and basal [Ca^2+^]_i_ under these conditions were similar across the 4 groups (Figure 2b, middle). Restoration of extracellular Ca^2+^ caused a transient overshoot of [Ca^2+^]_i_ in control and reverse-peptide treated astrocytes, and this was significantly suppressed in cells exposed to A*β*_1-42_. This difference aside, our results are not consistent with the idea that A*β*_1–42_ is toxic due to its ability to raise [Ca^2+^]_i_ as has previously been suggested.^20^

**Figure 3:**
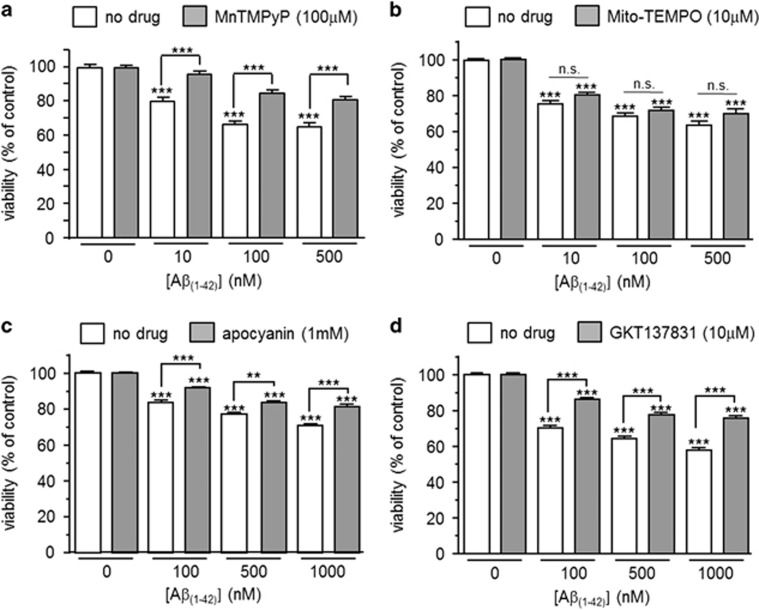
Evidence for the involvement of NADPH oxidase-derived ROS in A*β*_1–42_ toxicity. (**a**) Effect on cell viability of a 24 h exposure of astrocytes to A*β*_1–42_ alone (10–500 nM, white bars) or A*β*_1–42_ in the additional presence of 100 *μ*M MnTMPyP (grey bars). Bars represent the mean±S.E.M. data of cells from 3 repeats (each performed in duplicate) with cells from different passages. (**b**) as (**a**), except cells were either treated with A*β*_1–42_ alone (10–500 nM, white bars) or A*β*_1–42_ in the additional presence of 10 *μ*M Mito-TEMPO (grey bars). Bars represent the mean±S.E.M. data of cells from 3 repeats (each performed in duplicate). Significance: ***P<0.001 as compared with respective controls, or between cells without drug *versus* MnTMPyP (**a**). NS, not significant. (**c**) Effect on cell viability of a 24 h exposure of astrocytes to A*β*_1–42_ alone (100–1000 nM, white bars) or A*β*_1–42_ in the additional presence of 1 mM apocyanin (grey bars). Bars represent the mean±S.E.M. data of cells from 3 repeats (each performed in duplicate) with cells from different preparations. (**d**) as (**c**), except cells were either treated with A*β*_1–42_ alone (100–1000 nM, white bars) or A*β*_1–42_ in the additional presence of 10 *μ*M GKT137831 (grey bars). Bars represent the mean±S.E.M. data of cells from 2 repeats (each performed in duplicate) with cells from different preparations. Significance: **P<0.01; ***P<0.001 as compared either with respective controls, or between drug treatment or no treatment, as indicated

ROS generation has often been associated with amyloid toxicity.^25, 26^ To investigate any potential role for ROS in amyloid-mediated loss of astrocyte viability, we first examined the effects of MnTMPyP, a superoxide dismutase mimetic. As shown in Figure 3a, MnTMPyP significantly ameliorated the toxic effects of A*β*_1–42_. However, treatment of cells with mito-TEMPO, a mitochondria targeted antioxidant, was unable to modify significantly the toxic effects of A*β*_1–42_, suggesting that ROS were not derived from mitochondria (Figure 3b). Instead, a significant reduction in the toxicity of A*β*_1–42_ was observed in the presence of either apocyanin, a non-specific inhibitor of NADPH oxidase (Figure 3c), or the NOX1/NOX4 inhibitor, GKT137831^27^ (Figure 3d). These data are consistent with NADPH oxidase as being at least partly responsible for the production of toxic ROS following exposure to A*β*_1–42_.

**Figure 4:**
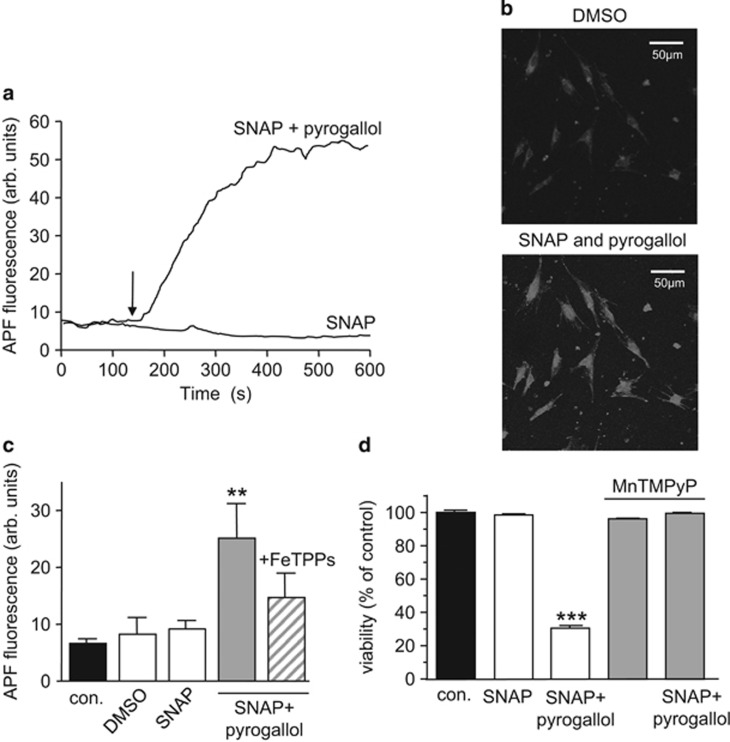
Peroxynitrite is highly toxic to astrocytes. (**a**) Fluorescence images of astrocytes loaded with the ONOO^–^ sensitive dye, 2-[6-(4′-amino) phenoxy-3H-xanthen-3-on-9-yl]benzoic acid (APF). Cells were first treated with vehicle (DMSO; 1:1000) and then SNAP (200 *μ*M) alone or together with pyrogallol (100 *μ*M). (**b**) Example timecourses of the changes in APF fluorescence seen in cells exposed to SNAP alone (10 *μ*M) or SNAP together with pyrogallol (100 *μ*M). (**c**) Mean±S.E.M. peak fluorescence detected in astrocytes exposed to vehicle, SNAP (200 *μ*M), SNAP together with pyrogallol (100 *μ*M) or both agents together with the ONOO^–^ scavenger FeTPPS (50 *μ*M). **P<0.01 as compared with control. (**d**) Effect on cell viability of a 24 h exposure of astrocytes to SNAP (200 *μ*M), SNAP together with pyrogallol (100 *μ*M) or both agents together with the antioxidant MnTMPyP (100 *μ*M). Each bar represents the mean±S.E.M. taken from between 4 and 8 observations. Significance; ***P<0.001 as compared with control

It was noteworthy in the studies reported in Figures 2 and 3 that ROS inhibition could not fully reverse the toxic effects of A*β*_1–42_, suggesting the involvement of other factors. Amyloid peptides have long been known to increase nitric oxide (NO) formation via induction of iNOS in astrocytes and neurones.^24, 28, 29, 30^ Increased formation of NO, in the presence of elevated ROS levels, can lead to formation of the highly toxic ROS, peroxynitrite (ONOO^**–**^). To explore a possible role for ONOO^–^ we first examined its effects on astrocyte viability. ONOO^–^ formation was monitored using 2-[6-(4′-amino) phenoxy-3H-xanthen-3-on-9-yl]benzoic acid (APF) fluorescence. Exposure of cells to the NO donor S-nitrosopenacillamine (SNAP; 200 *μ*M) alone was without effect on APF fluorescence (Figure 4a), but when applied with pyrogallol (100 *μ*M), which auto-oxidises to form superoxide, a clear rise of APF fluorescence was apparent (Figures 4a–c). Figure 4b illustrates two images of APF fluorescence, before and during exposure to SNAP together with pyrogallol. Mean data are plotted in Figure 4c, which also illustrates the ability of the ONOO^–^ scavenger FeTPPs (5,10,15,20-tetrakis-[4-sulfonatophenyl]-porphyrinato-iron[III] 50 *μ*M), which converts ONOO^–^ rapidly to nitrate,^31^ to reduce the SNAP / pyrogallol rise of ONOO^–^. Figure 4d shows that ONOO^–^ is highly toxic to astrocytes: exposure to SNAP alone was without significant effect on astrocyte viability, but together with pyrogallol it caused a striking loss of cells. This effect was prevented by the superoxide dismutase mimetic MnTMPyP (Figure 4d).

**Figure 5:**
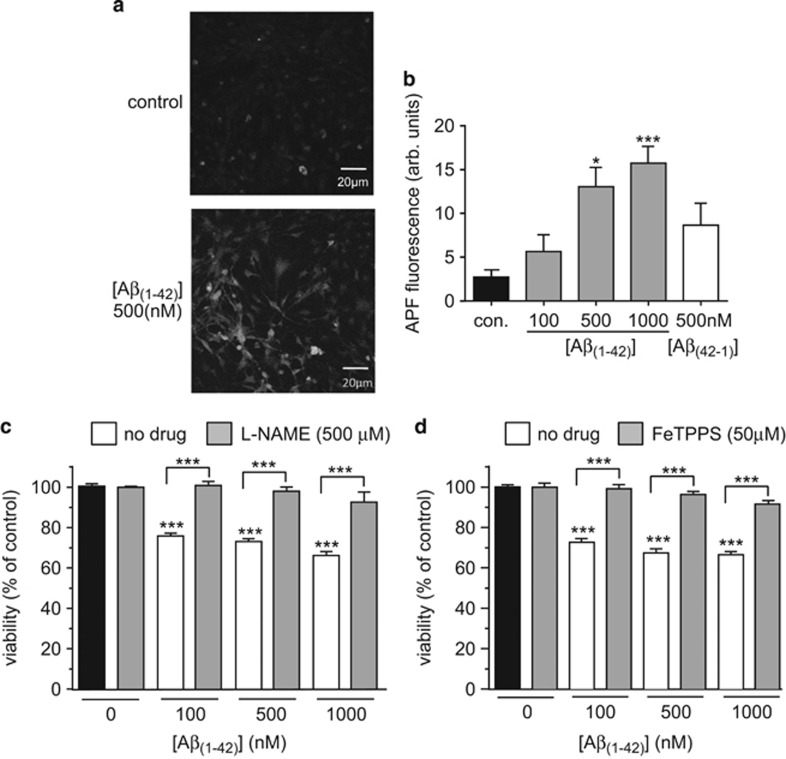
A*β*_1-42_toxicity involves peroxynitrite formation. (**a**) Separate fluorescence images of astrocytes loaded with the ONOO^–^ sensitive dye, 2-[6-(4′-amino) phenoxy-3H-xanthen-3-on-9-yl]benzoic acid (APF). Cells were either untreated or exposed to 500 nM A*β*_1-42_ for 24 h. (**b**) Mean±S.E.M. (taken from 5-10 recordings) APF fluorescence detected in astrocytes without treatment, or following a 24 h treatment with either 100–1000 nM A*β*_1–42_ or reverse peptide (500 nM), as indicated. *P<0.05; **P<0.01 as compared with control. (**c**) Effect on cell viability of 24 h exposure of astrocytes to A*β*_1–42_ alone (100–1000 nM, white bars) or A*β*_1–42_ in the additional presence of 500 *μ*M L-NAME (grey bars). Bars represent the mean±S.E.M. data of cells from 6 repeats (each performed in duplicate) with cells from different preparations. Significance: ***P<0.001 as compared either with respective controls, or between drug treatment or no treatment, as indicated. (**d**) as (**c**), except that cells were exposed either to A*β*_1–42_ alone (100–1000 nM, white bars) or A*β*_1–42_ in the additional presence of 50 *μ*M FeTPPs (grey bars). Bars represent the mean±S.E.M. data of cells from 3 repeats. Significant difference: ***P<0.001 effects of peptide alone compared to control, or between amyloid peptide with or without FeTPPS at each amyloid concentration, as indicated

The data in Figure 4 show clearly that formation of ONOO^–^ in astrocytes is detectable, and is highly toxic. To investigate whether A*β*_1–42_ can exert its toxic actions through the formation of ONOO^–^ we first examined its ability to increase APF fluorescence. As exemplified in the images of Figure 5a and the mean data of Figure 5b, APF fluorescence was indeed increased following a 24 h exposure to A*β*_1–42_. Consistent with the idea that ONOO^–^ may contribute to its toxicity, we also found that A*β*_1–42_ induced loss of astrocyte viability was essentially completely prevented in the presence of L-NAME to prevent NO formation (Figure 5c). Similarly, in the presence of the ONOO^–^ scavenger FeTPPS, A*β*_1–42_ was without significant effect on astrocyte viability (Figure 5d. Together, these findings suggest that A*β*_1–42_ is deleterious to astrocytes due to stimulation of elevated levels of both ROS and NO, which subsequently form ONOO^−^.

**Figure 6:**
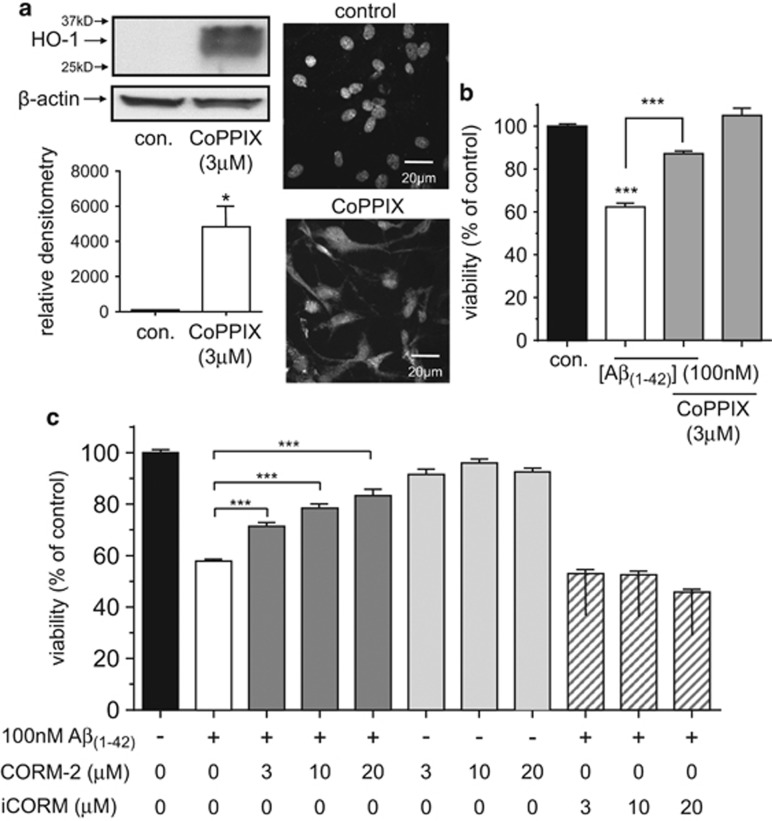
HO-1 induction protects astrocytes from A*β*_1–42_ toxicity via CO formation. (**a**) Left, western blot for HO-1 taken from control astrocytes and astrocytes exposed to CoPPIX (3 *μ*M) for 24 h, as indicated. *β*-actin was also probed to confirm approximately equal protein loading of lanes. Below, mean±S.E.M. (*n*=3) relative densitometric readings for control and CoPPIX-treated cells, as indicated. *P<0.05. Right, images of control and CoPPIX-treated cells, immunostained for HO-1. Scale bar applied to both images. (**b**) Effect on cell viability of a 24 h exposure of astrocytes to A*β*_1–42_ alone (100 nM, white bar) or A*β*_1–42_ in the additional presence of 3 *μ*M CoPPIX. Also shown is the lack of effect of CoPPIX alone. Bars represent the mean±S.E.M. data of cells from 5 repeats (each performed in duplicate) with cells from different preparations. ***P<0.001. (**c**) Effect on cell viability of a 24 h exposure of astrocytes to 100 nM A*β*_1–42_ in the absence (white bar) or presence (dark grey bars) of the CO donor CORM-2 (3–20 *μ*M). The effects of CORM-2 alone are also presented (light grey bars), along with the effects of 100 nM A*β*_1–42_ in the additional presence of the inactive form of CORM-2, iCORM (hatched bars). Bars represent the mean±S.E.M. data of cells from 6 repeats (each performed in duplicate) with cells from different preparations. ***P<0.001

We have previously shown that induction of HO-1 affords protection in neurons against the toxicity of A*β*_1–42._^32^ To investigate whether HO-1 was similarly protective in astrocytes, we first induced its expression by exposing astrocytes to an HO-1 inducer, cobalt protoporphyrin IX (CoPPIX; 3 *μ*M) for 24 h. As shown in Figure 6a such treatment caused a strong induction of HO-1, and in CoPPIX-treated astrocytes, the toxic effects of A*β*_1–42_ (added for the same 24 h exposure to CoPPIX) were significantly attenuated (Figure 6b). Earlier studies have shown that carbon monoxide (CO), a product of HO-1-mediated haem degradation, can provide protection against apoptosis^33, 34^ and so we investigated such a role for CO in astrocytes. As illustrated in Figure 6c, exposure of cells to the CO donor CORM-2 (3–20 *μ*M) caused a concentration-dependent reversal of A*β*_1–42_ toxicity without significantly affecting the viability of astrocytes not exposed to A*β*_1–42_. The control compound iCORM (which does not release CO) was unable to affect the toxicity of A*β*_1–42_. Neither CORM-2 nor iCORM induced significant levels of HO-1 themselves (Supplementary Figure 1). Similarly, L-NAME did not alter HO-1 expression significantly (Supplementary Figure 1). Biliverdin, another HO-1 product, was without effect on the toxicity of A*β*_1–42_ (Supplementary Figure 2).

**Figure 7:**
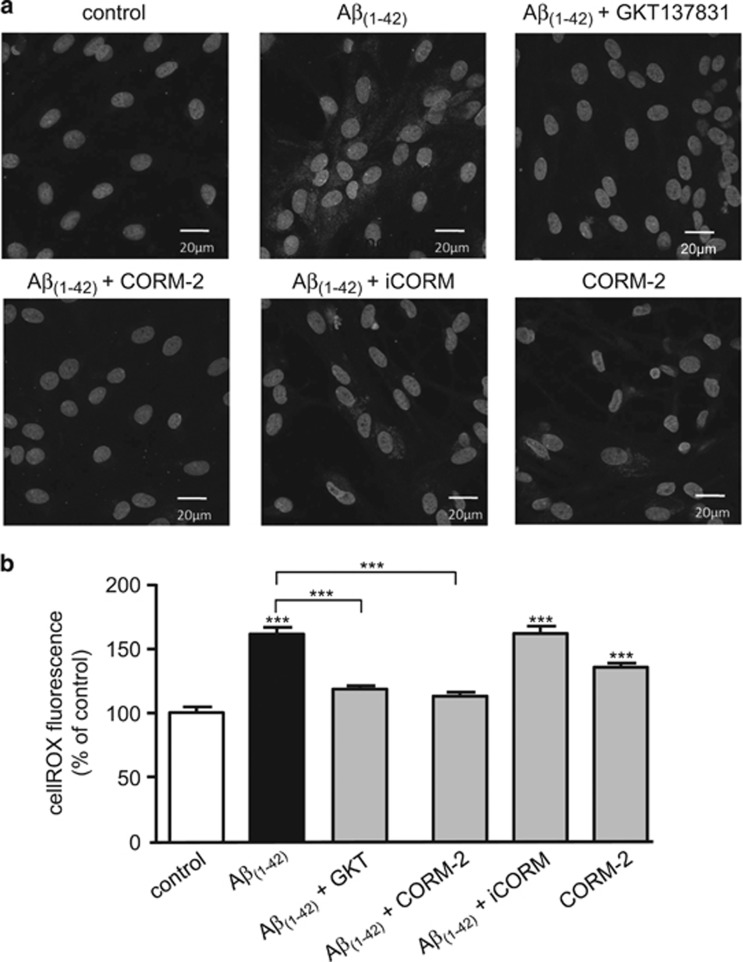
A*β*_1–42_ increases ROS formation from NADPH oxidase. (**a**) Representative CellROX images of astrocytes under control conditions, or following a 24 h treatment with 100 nM A*β*_1–42_ alone, or together with GKT137831 (10 *μ*M), or CORM-2 (20 *μ*M) or iCORM (20 *μ*M), as indicated. Bottom right image shows the effects of 20 *μ*M CORM-2 alone. All images also show DAPI (nuclear) staining. Scale bar applies to all images. (B) Mean±S.E.M. fluorescence determined under the conditions exemplified in **a**. Data taken from 10 regions of interest, measured in 3–5 images from 3 experimental repeats. Significance: ***P<0.001 as compared with control or compared with 100 nM A*β*_1–42_ treatment, as indicated

A previous study has reported that CO inhibits NADPH oxidase in proliferating smooth muscle.^35^ Since NADPH oxidase was a significant source of ROS mediating A*β*_1–42_ toxicity (Figure 3), we investigated whether CO inhibition of NADPH oxidase activity accounted for its protective effects against A*β*_1–42_ toxicity. To do this, we examined ROS formation using CellROX deep Red, a fluoroprobe which emits fluorescence upon oxidation. Representative images are shown in Figure 7a, and quantified in Figure 7b. As compared with untreated cells, those exposed to A*β*_1–42_ showed a significant increase in ROS production (increased cytoplasmic fluorescence) and this was significantly reduced by the NOX1/NOX4 inhibitor, GKT137831 (10 *μ*M). Fluorescence was also significantly suppressed by 20 *μ*M CORM-2 but not by iCORM. Interestingly, CORM-2 alone caused a modest rise in fluorescence, presumably because it is known to stimulate ROS formation from mitochondria.^36^

**Figure 8:**
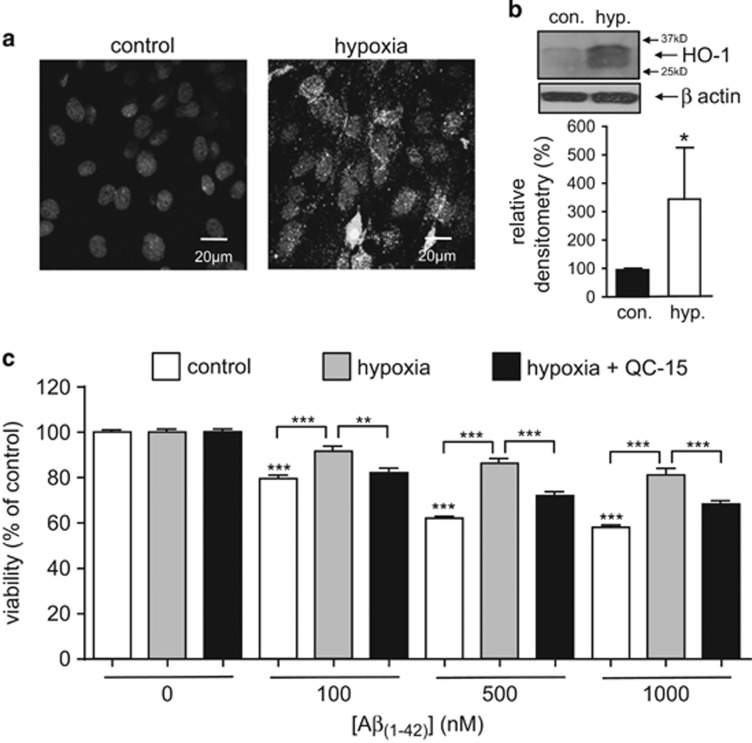
Hypoxia protects against A*β*_1–42_ toxicity via HO-1 induction. (**a**) Representative images of astrocytes immunostained for HO-1 under control conditions, or following a 48 h exposure to hypoxia (0.5% O_2_) as indicated. Scale bar applies to both images. (**b**) Example of western blot showing induction of HO-1 by hypoxia (hyp., 48 h, 0.5% O_2_). Bar graph plots mean±S.E.M. (*n*=3) densitometry (relative to control (normoxia)) taken from blots. *P<0.05. (**c**) Effect on cell viability of a 24 h exposure of astrocytes to A*β*_1–42_ alone (100–1000 nM, white bars) or A*β*_1–42_ under hypoxic conditions in the absence (grey bars) or additional presence of 10 *μ*M QC-15 (black bars). Bars represent the mean±S.E.M. data of cells from 3 repeats (each performed in duplicate) with cells from different preparations. Significance: **P<0.01; ***P<0.001 as compared with control or as compared between treatments, as indicated

HO-1 is induced by several forms of cellular stress, prominent amongst which is hypoxia.^37, 38^ We maintained astrocytes in hypoxia (0.5% O_2_) for 48 h and found this induced HO-1 strongly, as observed using both immunohistochemistry (Figure 8a) and western blotting (Figure 8b). Astrocytes maintained in hypoxia for a subsequent 24 h exposure to A*β*_1–42_ were significantly more resistant to toxicity than those maintained under control (normoxic) conditions (Figure 8c). Hypoxic resistance to A*β*_1–42_ toxicity appeared to be due specifically to HO-1 induction, since it was largely prevented by application of the selective HO-1 inhibitor QC-15^39^ (Figure 8c).

## Discussion

The present study demonstrates that sub-micromolar levels of A*β*_1–42_ have a significant impact on astrocyte viability over a 24 h period in comparison to a control peptide. Whilst these findings at least superficially agree with some previous studies,^24^ others have indicated that A*β*_1–42_ does not alter astrocyte viability,^23^ but can disrupt [Ca^2+^]_i_^20^. These latter findings contrast with our observations both on viability (Figure 1) and alterations in [Ca^2+^]_i_ (Figure 2). At present we cannot account for such different responses, but one likely possibility is the peptide preparation used. Our studies have employed peptides that were maintained for 24 h at 37 °C in medium before being applied to cells. We have previously shown that under these conditions cells are exposed to a mixture of monomers, small globular assemblies and protofibrils, as assessed by electron microscopy.^32^ By contrast, elevations in astrocytic [Ca^2+^]_i_ were observed following acute application of 10–50 *μ*M of A*β*_1–42_ which was prepared in ultrapure water, reducing the likelihood of aggregation.^40, 41^ Despite these reported differences, what is clear from this study and others is that A*β*_1–42_ can elevate astrocytic ROS levels. This is likely to be pathologically important, and precedes amyloid-induced increases of ROS in neurons.^42^ ROS derived from mitochondria have been implicated in ageing and associated with Ca^2+^ mobilisation in astrocytes.^43^ In the present study, NADPH oxidase(s) appear to be major contributors to the increased ROS levels (see ref. 44 and Figure 3). Although we have not explored the mechanism underlying amyloid-mediated stimulation of NADPH oxidase activity, it has previously been suggested that this occurs via Ca^2+^-dependent activation of protein kinase C*β*.^44, 45^ Whether or not the same process underlies NADPH oxidase activation as reported here is unclear, but it is noteworthy that no elevation of [Ca^2+^]_i_ was observed in the present study (Figure 2).

Astrocytes respond to pro-inflammatory signals with the release of toxic species, including ONOO^–^ (see refs 46, 47). It has previously been reported that astrocytes are relatively resistant to ONOO^–^ toxicity when applied exogenously or derived from iNOS.^48, 49^ Indeed, although NO modulates mitochondrial function in astrocytes, no overt toxicity has been reported^50^ in agreement with this study (Figure 4d). However, all three isoforms of nitric oxide synthase (NOS) are elevated in AD,^51, 52^ and Lipton and colleagues have produced a number of studies which collectively provide compelling evidence that much of the toxicity of amyloid peptides arises due to stimulation of NO production.^53, 54, 55, 56^ Aberrant, or excessive nitrosylation of target proteins (that is, conversion of cysteine –SH groups to –SNO groups) accounts for many of these deleterious effects.^30, 55, 56^ However, amyloid peptides have also been reported to increase ONOO^–^ levels *in vivo*^46, 47^ and this can also impact on protein function through nitration of tyrosine residues.^57^ Indeed, A*β*_1–42_ has been reported to be a target of nitration, which can result in increased peptide aggregation.^58^ Given these reported differential sensitivities to NO and NO-related ROS, we examined the involvement of both NO and ONOO^–^. Consistent with previous studies our data highlighted that NO alone does not mediate amyloid toxicity. However, when added with a source of superoxide, pyrogallol, a dramatic rise of ONOO^–^ was observed, together with a large reduction in cell viability (Figure 4). These effects could be reversed by the ONOO^–^ scavenger, FeTPPs, the superoxide dismutase mimetic MnTMPyP, and also by L-NAME-mediated inhibition of NO formation (Figures 3, 4 and 5). As we also demonstrated that A*β*_1–42_ could directly raise ONOO^–^ levels (Figure 5b), our data strongly suggest that the toxic effects of A*β*_1–42_ on astrocytes are due to its ability to promote both ROS and NO formation and hence increase ONOO^–^ levels.

The cytotoxic actions of ONOO^–^ have been linked to intracellular glutathione levels and also to haem oxygenase activity.^59^ Induction of HO-1 in both neurons and astrocytes is well known to be associated with AD^60, 61^ although whether this is beneficial, or contributes to disease progression, is subject to debate: HO-1 induction specifically in glia has been shown to be detrimental because of the oxidative activity of iron liberated by haem degradation.^62, 63^ However, we^32^ and others^64, 65^ have provided evidence that HO-1 induction is protective. In the present study, HO-1 induction (either chemically or via exposure to hypoxia) is clearly protective against the toxic effects of A*β*_1–42_ and this protection is attributable to the formation of CO. Similarly in neurons we have shown that CO is protective against A*β*_1–42_ toxicity, but significantly the underlying mechanisms are quite distinct: in neurons, protection was attributable to inhibition of AMP-dependent protein kinase activation.^32^ In contrast, the present study demonstrates that CO provides protection via inhibition of ROS production in astrocytes specifically by NADPH oxidase. This correlates well with observations in smooth muscle cells and macrophages, where HO-1 induction, and resultant CO formation suppresses NADPH oxidase activity.^35^ This result is perhaps surprising, given that CO can itself increase ROS formation (Figure 7), not from NADPH oxidase but from mitochondria.^36^ Furthermore, CO can increase formation of NO in various cell types (e.g. refs 66, 67) and can in some instances itself be damaging through formation of ONOO^–^, as shown in neuroblastoma (SH-SY5Y) cells.^66^ The source of ROS and / or the specific cell type is therefore likely to be key to outcomes. Clearly, the present study shows that CO is protective against A*β*_1–42_ toxicity in astrocytes, and this is mediated through suppression of NADPH oxidase activity.

In summary, we have shown that sub-micromolar concentrations A*β*_1–42_ are toxic to astrocytes, due to the activation of NADPH oxidase and subsequent elevation of ROS. In the presence of tonic NOS activity, ONOO^–^ formation is increased. Induction of HO-1 provides protection against A*β*_(__1–42__)_ toxicity primarily via inhibition of NADPH oxidase. Our findings therefore further support the idea that HO-1 / CO is protective in the central nervous system and reveals potential mechanisms by which neuroprotection may be enhanced in the face of A*β*_1–42_ cellular toxicity of AD.

## Materials and Methods

### Astrocyte preparation

To obtain primary cultures of astrocytes, cerebral cortices were removed from 5–7-day-old Wistar rats and placed in ice-cold phosphate-buffered solution (PBS) containing no Ca^2+^ or Mg^2+^ (Gibco, Thermo-Fisher, Paisley, UK). Meninges were removed and cortices were minced with a razor blade and dispersed into the same buffer containing 0.25 mg/ml trypsin, at 37 °C for 15 min. Trypsin digestion was halted by the addition of an equal volume of buffer supplemented with 16 *μ*g/ml soy bean trypsin inhibitor (type I-S; Sigma Aldrich (Gillingham, UK), UK), 0.5 *μ*g/ml DNase I (EC 3.1.21.1 type II from bovine pancreas; 125 kU/ml; Sigma Aldrich) and 0.3 mM MgSO_4_. The digested tissue was then pelleted by centrifugation at 400 × *g* for 5 min and the supernatant decanted before resuspending the cell pellet in 6.8 ml of buffer solution containing 100 *μ*g/ml soy bean trypsin inhibitor, 0.5 *μ*g/ml DNase I and 1.5 mM MgSO_4_. Tissue was subsequently triturated gently with a 10 ml stripette (10 ×). The cloudy cell suspension was pipetted into 120 ml of media. The culture medium consisted of Eagle’s minimal essential medium supplemented with 10% foetal calf serum (v/v) and 1% (v/v) penicillin-streptomycin (Invitrogen, Paisley, UK). The cell suspension was then aliquoted into 75 cm^2^ flasks. Cells were then kept in a humidified incubator at 37 °C (95% air, 5% CO_2_). Six hours after plating out the cell suspension, cells were washed with fresh media to remove non-adhered cells and debris. This resulted in a culture of astrocytes (GFAP positive) as previously described.^68, 69^ Culture medium was exchanged every 7 days and cells were grown in culture for up to 14 days.

### MTT assays

Cell viability was investigated using MTT assays, as previously described.^32^ This technique compares well with the ATP-based CellTiter-Glo Luminescent Cell Viability Assay (Promega; SI Figure 3). Cells were cultured in poly-lysine coated 96-well plates to ~50% confluence or greater. Experiments were only carried out when all of the cell groups showed a similar confluency when viewed under the microscope. The final volume of each well after any treatment was kept at 100 *μ*l. Cells were treated for 24 h with different concentrations of either A*β*_1–42_ or the reverse peptide A*β*_42-1_ made up in serum-free media (SFM). The media in the control cells was also replaced with SFM for 24 h to ensure that all observations made were due to the application of A*β* rather than the result of serum withdrawal. This was done for all the experiments involving A*β* application.

When applying the tricarbonyldichlororuthenium(II) dimer (CORM-2) for 24 h, cells were treated twice a day (0930am and 1700 hours) to replenish the amount of CO in the media. Some cells were treated in parallel identically with iCORM, the inactive, control compound which cannot release CO. Following the 24 h treatments with A*β* and CORM-2, the media was discarded and the cells washed gently with PBS. This step was repeated to get rid of all the CORM-2 as it reacts with the MTT. Then the PBS was replaced with 100 *μ*l of fresh cell culture media in each well. For the MnTMPyP experiments, the cells were pre-treated with MnTMPyP for 30 min prior to applying A*β*_1–42_. For the L-NAME experiments, the cells were pre-treated with L-NAME for 1 h prior to treating with A*β*_1–42_. Next, 11 *μ*l of Thiazolyl Blue Tetrazolium Bromide (5 mg/ml, MTT, Sigma) made up in sterile PBS was added to each well (10% by volume) and the cells were incubated at 37 °C for 3 h. An equal volume (111 *μ*l per well) of solubilizing solution consisting of isopropanol and HCl (24 ml propan-1-ol/isopropyl alcohol (Sigma) + 1 ml 1M HCl) was added to each well to lyse the cells and the contents of each well was thoroughly mixed by pipetting. Absorbance was measured at 570 nm and at 630 nm using a spectrophotometer. The experiments were done in duplicate and repeated using cells from at least 3 different rat preparations to ensure the reliability of results. All of the results were normalised to untreated control cells and shown as a percentage change in cell viability compared to the corresponding controls.

### APF fluorescence

APF (2-[6-(4′-amino) phenoxy-3H-xanthen-3-on-9-yl]benzoic acid) fluorescence was used to detect peroxynitrite (ONOO^–^) formation as previously described.^66^ Cells were plated on to coverslips in 24-well plates and when needed for experiments coverslips containing cells were incubated with 100 nM, 500 nM, 1 *μ*M A*β*_1–42_ or 500 nM A*β*_42-1_ for 24 h. Following the 24 h treatments the cells were incubated with 10 *μ*M APF (Sigma Aldrich, UK) made up in HEPES buffered saline and incubated in the dark for 1 h at 37 °C. Following the 1 h incubation period, the coverslip was cut into fragments and one fragment was placed on a glass slide containing 200 *μ*l of HEPES- buffered saline with 10 *μ*M APF. APF was used due to its limited nonselective reactivity and resistance to light-induced auto-oxidation. Oxidation causes bright green fluorescence with an excitation/emission maxima of around 490/515 nm. Fluorescence increases when APF reacts with ONOO^–^ and the changes in fluorescence intensity were measured using a ZEISS (Oberkochen, Germany) laser scanning confocal microscope (LSM 510). The change in fluorescence was measured continuously for a total of 10 min. The fluophore was excited at 488 nm (emission was at 510 nm) by sequential scanning with argon lasers and the Zeiss AIM software was used to obtain the images. The same brightness, contrast and gamma settings were used for each condition.

### Western blotting

Cells used for immunoblotting were cultured in T75 flasks and when confluent, were treated with A*β*_1–42_, A*β*_42-1_ or cobalt protoporphyrin (CoPPIX) at the concentrations indicated in the Results for 24 h. For the hypoxic experiments, cells were exposed to hypoxia (0.5% O_2_) for 48 h. Following the treatments, cells were washed in PBS and then lysed *in situ* with 600 *μ*l of mammalian protein extraction reagent (M-PER, Pierce) containing complete protease inhibitor tablets (Roche) for 30 min at room temperature. Protein levels in the lysates were assessed using a BCA assay (Pierce). Cell proteins (typically 30 *μ*g protein per lane) were separated on 12.5%, 0.75 mm thick polyacrylamide SDS gels and electrophoretically transferred onto 0.2 *μ*m PVDF membranes (BioRad). The blots were blocked for 1 h with 10% milk protein in Tris-buffered saline with 0.05% Tween (TBST) then probed with primary antibody raised against HO-1 (1:200, rabbit polyclonal, Santa Cruz technologies or 1:1000 rabbit polyclonal, GeneTEX) at 4 °C overnight. Next, membranes were washed with TBST for 3 × 10 min prior to incubating with anti-rabbit horse radish peroxidise-conjugated secondary antibody (1:2000; Amersham Pharmacia Biotech, Buckinghamshire UK) for 1 h at room temperature. Following this incubation, membranes were washed in TBST for 3 × 10 min and bands visualised using an enhanced chemiluminescence detection system and hyperfilm ECL (Merck, UK).

### Immunofluorescence

Cells were cultured on poly-lysine coated glass coverslips in 6-well plates at >50% confluence prior to treatment with A*β*_1–42_, A*β*_42-1_ or cobalt protoporphyrin (CoPPIX) at the concentrations indicated in the Results for 24 h, or prior to exposure to hypoxia (0.5% O_2_, 48 h). Following said treatments, cells were immunostained for HO-1 expression. Briefly, media was discarded and the cells were washed (3 × 5 min) with Dulbecco’s PBS. Cells were then fixed with paraformaldehyde (4% in PBS) for 20 min, following which they were permeabilized with PBS containing 0.22% Triton X100 supplemented with 10% normal goat serum (NGS; Sigma). Following 3 × 5 min washes with Dulbecco’s PBS containing 1% NGS, cells were then incubated overnight at 4 °C with the primary antibody; rabbit polyclonal anti-HO-1 (1:100, Santa Cruz) in Dulbecco’s PBS containing 1% NGS. The following day, cells were washed with Dulbecco’s PBS containing 1% NGS (3 × 5 min). Antibody binding was visualised by incubating the cells with a secondary antibody; Alexa Fluor-488 conjugated anti-rabbit IgG (1:1000, Invitrogen), for 1 h in the dark. Post-incubation, and following 3 × 5 min washes with Dulbecco’s PBS, coverslips were mounted on slides using Vectashield^R^ mounting media containing DAPI (Vector Laboratories, CA). The slides were then examined using a Zeiss laser scanning confocal microscope (LSM 700).

### Amyloid beta preparation

A*β*_1–42_ and A*β*_42-1_ (r-Peptides, Bogart USA) were dissolved in DMEM (Gibco, Paisley, UK) to make up 100 *μ*M stock solutions and kept at −20 °C. In order to form protofibrils prior to treating the cells, the A*β* peptide was maintained at 37 °C for 24 h, as previously described.^32^

### CellROX assay

Cells were cultured on poly-lysine coated glass coverslips in 6-well plates at ⩾50% confluence prior to treatment as described in the Results section. Following treatment the media was removed, cells were washed with PBS and 5 *μ*M CellROX deep red regent (Molecular Probes, Life Technologies, Paisley, UK) was applied for 30 min in the dark at 37 °C. Thereafter, cells were washed three times with PBS and fixed with 10% buffered formaldehyde (Sigma) for 15 min. Cells were then washed with PBS and the coverslips were mounted on slides using Vectorshield mounting media containing DAPI (Vector Laboratories, Burlingame, CA, USA). Slides were then examined using a Zeiss laser scanning confocal microscope (LSM 700). Images for all the treatments on a particular day were taken using identical settings. ImageJ software (NIH, Bethesda, USA) was used to analyse the images. To do this, 10 regions of interest were obtained for each image and 3–5 images each were taken for any given treatment on any given experimental day.

### Statistical analysis

Data are shown as mean±S.E.M. Statistical analysis was carried out using one-way ANOVA followed by either the Dunnett’s or Bonferroni *post test*, as appropriate. *P*-values of <0.05 were considered significant. CellROX results were analysed using a two-way ANOVA followed by a Bonferroni *post test*. *P*<0.05 was considered to be significant.
