# Supplementary material for: Heme oxygenase-1 derived carbon monoxide suppresses Aβ1–42 toxicity in astrocytes
Source: Cell Death Dis. 2017 Jun 15;8(6):e2884–. doi: 10.1038/cddis.2017.276 (PMC5520931; doi:10.1038/cddis.2017.276)
Supplement: Supplementary Figures [file cddis2017276x1.ppt]

## Slide 1
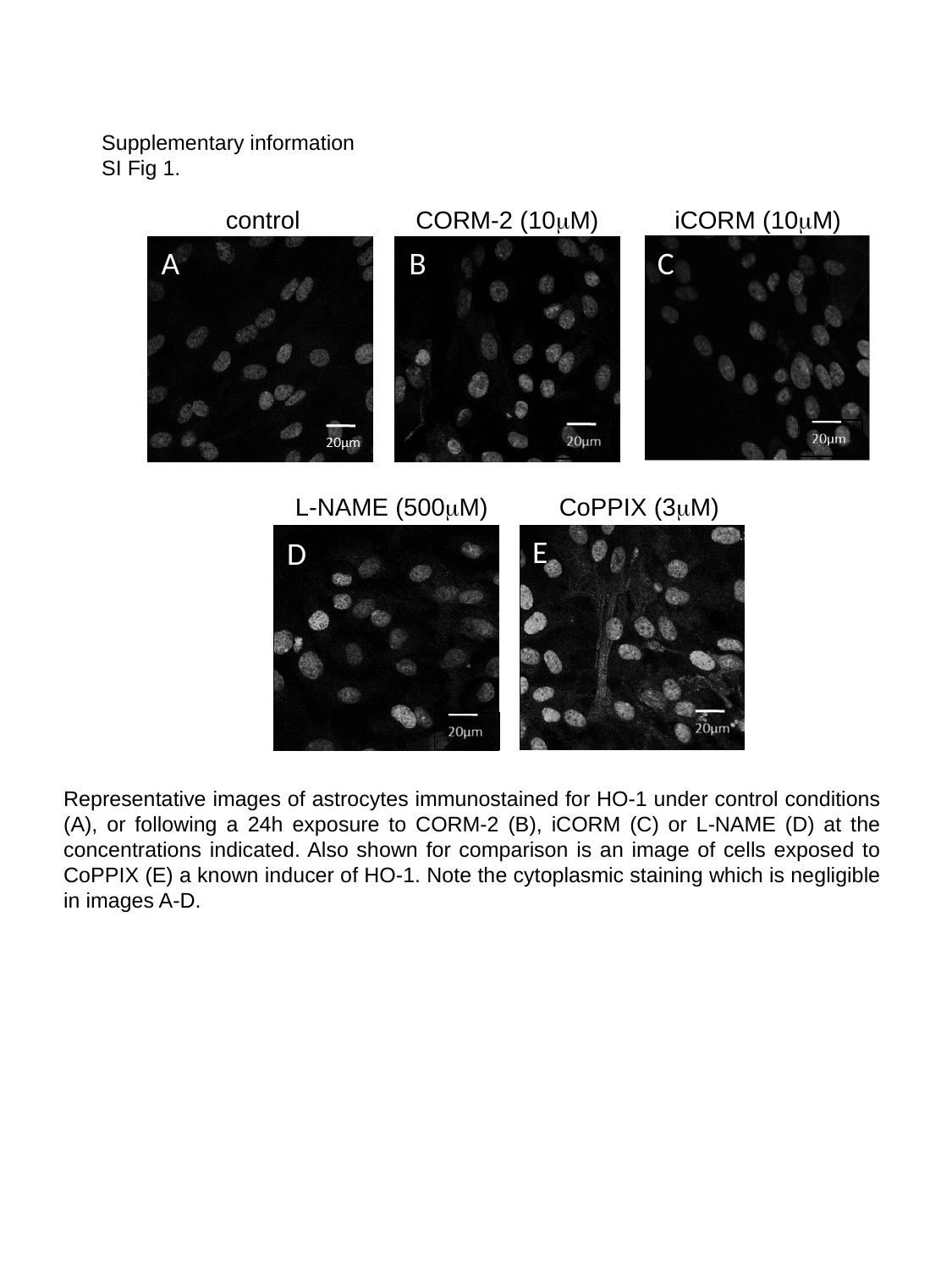

Supplementary information
SI Fig 1.
control
CORM-2 (10μM)
iCORM (10μM)
A
C
B
CoPPIX (3μM)
L-NAME (500μM)
E
D
Representative images of astrocytes immunostained for HO-1 under control conditions (A), or following a 24h exposure to CORM-2 (B), iCORM (C) or L-NAME (D) at the concentrations indicated. Also shown for comparison is an image of cells exposed to CoPPIX (E) a known inducer of HO-1. Note the cytoplasmic staining which is negligible in images A-D.

## Slide 2
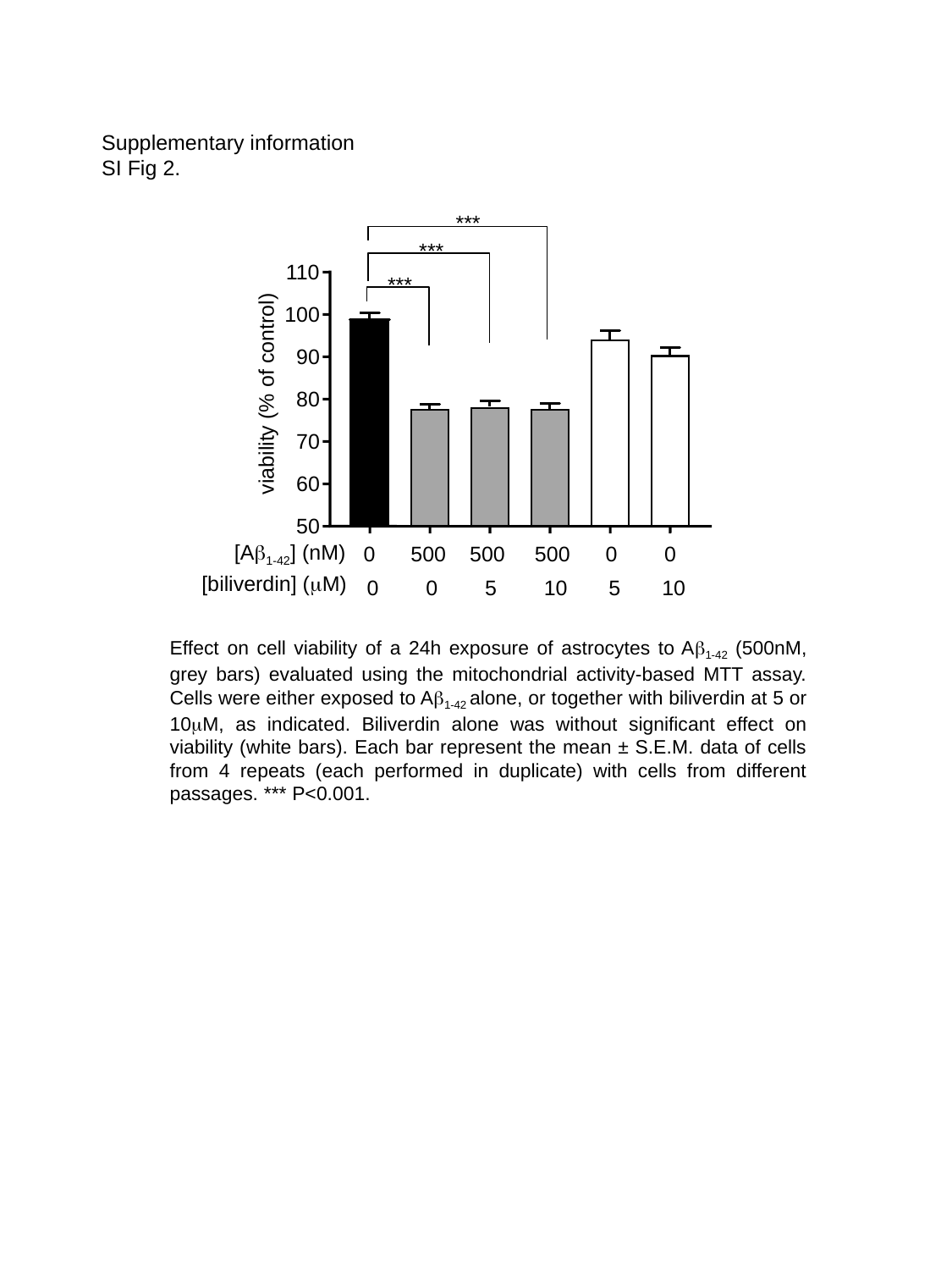

Supplementary information
SI Fig 2.
***
***
110
***
100
90
viability (% of control)
80
70
60
50
[Aβ1-42] (nM)
0 500 500 500 0 0
[biliverdin] (μM)
0 0 5 10 5 10
Effect on cell viability of a 24h exposure of astrocytes to Aβ1-42 (500nM, grey bars) evaluated using the mitochondrial activity-based MTT assay. Cells were either exposed to Aβ1-42 alone, or together with biliverdin at 5 or 10μM, as indicated. Biliverdin alone was without significant effect on viability (white bars). Each bar represent the mean ± S.E.M. data of cells from 4 repeats (each performed in duplicate) with cells from different passages. *** P<0.001.

## Slide 3
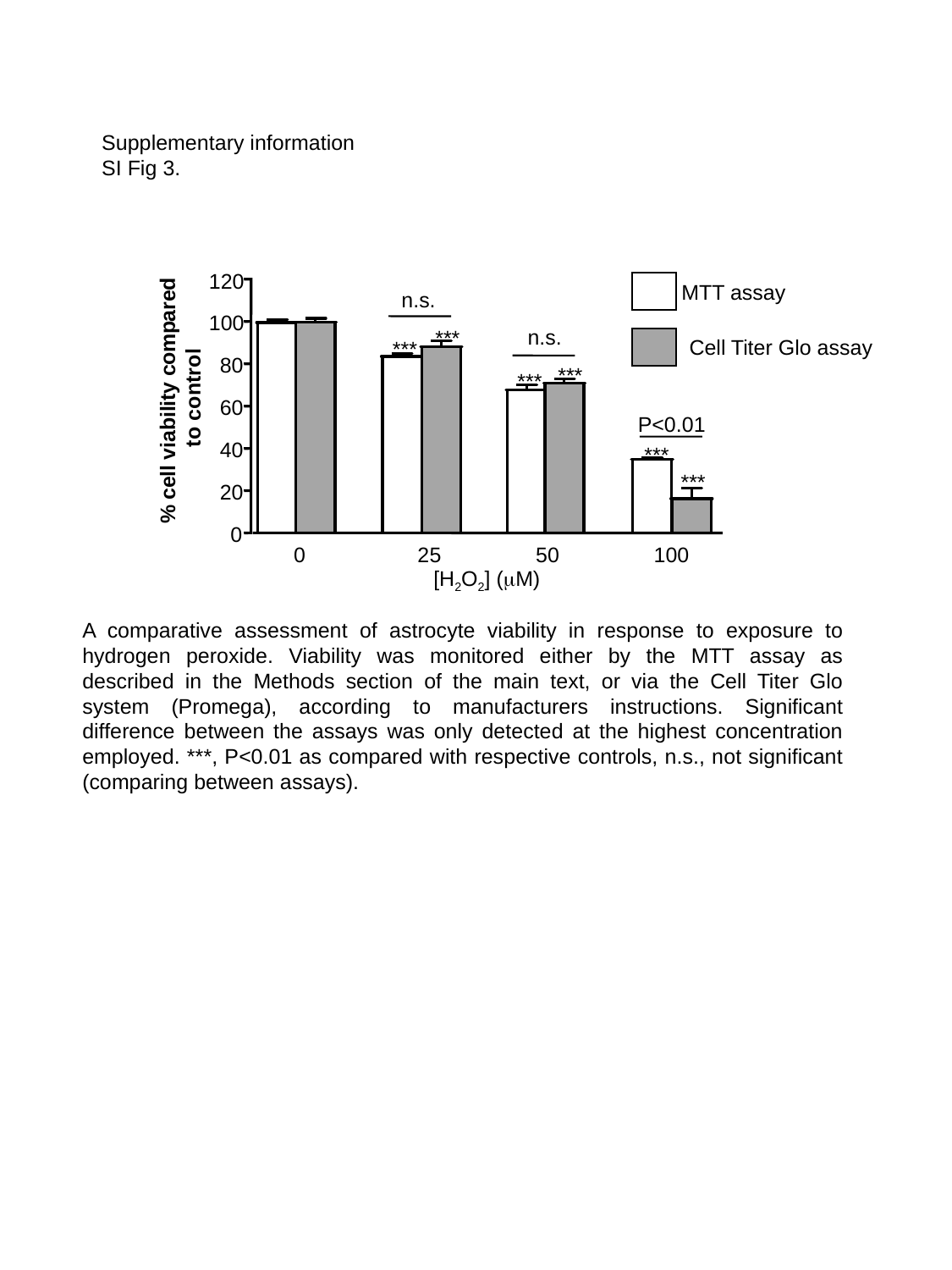

Supplementary information
SI Fig 3.
120
d
MTT assay
n.s.
e
r
a
100
p
n.s.
***
Cell Titer Glo assay
m
***
l
o
o
80
c
r
***
t
***
y
n
t
o
i
60
l
i
c
P<0.01
b
o
a
t
i
40
v
***
l
l
***
e
20
c
%
0
0 25 50 100
[H2O2] (μM)
A comparative assessment of astrocyte viability in response to exposure to hydrogen peroxide. Viability was monitored either by the MTT assay as described in the Methods section of the main text, or via the Cell Titer Glo system (Promega), according to manufacturers instructions. Significant difference between the assays was only detected at the highest concentration employed. ***, P<0.01 as compared with respective controls, n.s., not significant (comparing between assays).
